# Intestitial Nephritis*Read before the Nebraska State Medical Society, at Omaha, May, 11, 1892.

**Published:** 1892-10

**Authors:** W. O. Bridges

**Affiliations:** Omaha, Neb., Professor of Clinical Medicine, Omaha Medical College


### Southern Medical Record.

A MONTHLY JOURNAL OF MEDICINE AND SURGERY.

VOL. XXII. ATLANTA, GA., OCTOBER, 1892. NO. 10

### Original Articles.

### INTERSTITIAL NEPHRITIS.*

BY W. O. BRIDGES, M. D., OMAHA, NEB.,
Professor of Clinical Medicine, Omaha Medical College.

In selecting the above subject for a paper, I have done so with a view to call your attention to a disease which, experience has led me to believe, passes through its earlier stages unrecognized to a greater extent than any disease with which I am familiar, and also to insist upon the simplicity of its relatively early diagnosis, and the means at our command for its timely management.

By interstitial nephritis is meant an inflammation of the connective tissue of the kidney, always chronic in character, and in accordance with the unvarying law of connective tissue proliferation, leading to contraction of the organ, and consequent atrophy of the excreting apparatus ; hence, occasioning what is known as the cirrhotic or contracted kidney. I wish here to emphasize the discrimination between interstitial inflammation of the kidney, occurring *de novo*, and as a sequel of chronic parenchymatous nephritis. It is with the former

*Read before the Nebraska State Medical Society, at Omaha, May, 11, 1892.

that we have to deal. I stated that in a large proportion of cases, this disease passes unrecognized until the manifestations of its latter stages appear, and the probable reason is found in the want of uniformity of its early symptomatology, and the absence of such indications pointing to kidney disease, as usually lead us to examine the urine. To illustrate, I will briefly cite a few cases which have come under my observation in the past few years:

CASE I. H. N., aged 28, was a clerk by occupation; he had had scarlet fever in childhood, recovering without known complication or sequel. He had not been in robust health, and was occasionally troubled with an eczematous eruption of the hands. He had consulted physicians several times during the preceding year or two, for debility, depression of spirits without known cause, and had taken tonics with temporary benefit. The preceding winter he had complained of shortness of breath on exertion, but this was attributed to general debility. Suddenly one night, he was seized with an asthmatic paroxysm followed by cough and bloody expectoration. This was repeated on the two following nights, each being relieved by dry cupping over the chest. Before the third attack he felt able to attend to his duties in the day time, but following this one, he had a temperature of 101 degs., and fine moist rales were manifest over the entire chest. The countenance was pale, lips colorless, no œdema. An examination of the urine for the first time, showed a specific gravity of 1007, and a trace of albumen. On close questioning, he stated that during the past one and one-half years, he had been in the habit of rising earlier than usual to urinate, and latterly, the amount had increased considerably. There was no œdema, no dyspeptic symptoms, no headache. The heart was markedly enlarged, the apex beat being an inch below and to the left of the normal. There were no murmurs, but the second sound was much accentuated. A diagnosis of interstitial nephritis was made. From this time until his death, six months later, the chief symptoms were referable to the respiratory system; paroxysms of asthma mostly at night, followed by two or three days of pulmonary congestion and œdema, severe dyspnœa, attacks of œdema of the glottis of very short duration, and always terminating in intense swelling of the soft tissues about the larynx. At these times, suffocation appeared imminent, and was relieved only after hypodermics of pilocarpine.

Frequent examinations of the urine, showed albumen in varying quantities; always slight, specific gravity never reaching above 1009; amount from two to three quarts daily, very pale in color. Occasionally there was slight œdema of the feet and eyelids, and towards the last he had nausea and some vomiting. He died in uræmic coma.

CASE II. Was a grain dealer, 40 years of age, who had contracted a venereal sore in his early manhood, which was pronounced specific, and which disappeared under mercurial treatment kept up for only a few months. There were no secondary developments. He had a resilient stricture of the deep urethra, which necessitated the use of the sound once a month; this had been resorted to for many years. For a year previously he had been troubled with periodic headaches, which were followed by slight dyspeptic symptoms, and he had lost thirty pounds in weight. Latterly, the headaches had become more frequent and severe, and he had consulted several medical men, who pronounced his attacks bilious in character, and treated him accordingly, with temporary improvement. His headaches were peculiar, occurring always at four o'clock in the morning, and becoming so severe that he was obliged to rise and pace the floor until his servant could prepare very strong coffee, which afforded sufficient relief to enable him to rest again. By noon of the same day, it disappeared and left him weak and with slight nausea. The attacks recurred weekly on the same day at the time he consulted me, and I was inclined to the diagnosis of malarial infection. He was therefore given calomel and large doses of quinine preceding the expected attacks, which served only to postpone them, the effect probably being from calomel. A more thorough examination now revealed slight œdema at the ankle, and the urine was pale in color, specific gravity 1009, no albumen. A subsequent examination showed a trace of albumen; quantity in twenty-four hours, three quarts. All treatment gave only slight relief to the headaches; some of the seizures were so severe as to necessitate chloroform anæsthesia.

He was growing worse as the fall advanced, and on my advice, he went to Southern California. Here he remained until the following May, having only one attack soon after his arrival in San Diego. He improved in every way, except he did not regain his flesh. He returned here to his work apparently well. Urine still increased in quantity, specific gravity varied from 1011 to 1013, only occasional traces of albumen; no œdema, no dyspepsia. Improvement continued dur- the summer; as the fall advanced his headaches returned, and in spite of the warning given by their increasing severity and frequency and my advice, he delayed returning to the sunny clime until December. On the way, his train was delayed by severe storms in the mountains, he took cold and developed uræmic convulsions, which continued irregularly until he died, several weeks later in coma.

CASE III. L. K., a female servant, aged 27; had not been well for two years, during which time she had been treated by several physicians for heart disease and bladder trouble. Symptoms of bladder irritability had been manifest in frequent desire to urinate for many months, and latterly the urine was always cloudy. She had very slight puffiness of the lower eyelids, and there was occasional œdema of the feet. Nausea and vomiting occurred frequently, and at times the stomach would not tolerate any kind of food for several days. The heart was much enlarged, and a mitral systolic murmur detected. The urine was increased to over two quarts in twenty-four hours, and contained so much sediment consisting of pus and stringy mucus, that it was necessary to filter each specimen before the urinalysis could be completed. The color was pale, specific gravity 1005, albumen in very small quantity, large hyaline casts were found under the microscope. A diagnosis of cystitis, interstitial nephritis, and mitral insufficiency, was made. She has been under my observation for nearly two years.

Rapid improvement followed treatment of the cystitis, and she resumed housework. Several examinations of the urine have shown a specific gravity varying from 1002 (which was found more than once) to 1007, and always a trace of albumen.

In the absence of any rheumatic history, I considered the heart trouble secondary to an interstitial nephritis.

CASE IV. M. A., aged 27, had been a sufferer from dyspeptic trouble for two years. She had lost flesh and was in very depressed spirits. Diphtheria in childhood without known sequel was the only illness reported previously. All treatment had been directed to her stomach difficulty, and she was discouraged at its failure to relieve. There was no œdema, the heart was enlarged without valvular lesions. She excreted three quarts of urine in twenty-four hours. It was pale, specific gravity 1009, and contained albumen in small quantity. Several subsequent examinations gave about the same result. A consultation with an eastern physician of note corroborated a diagnosis of interstitial nephritis, and she was advised to remain in southern California one year. There she improved rapidly, her dyspeptic trouble disappeared, and in nine months she had gained twenty pounds, when she returned home. The urine was still increased in quantity, was pale, specific gravity 1015, no albumen. In the past year and a half she has lost and gained alternately; lost during her stay at home and gained when in a warm climate. Her principal complaint is of dyspepsia and depesssion of spirits. Occasional traces of albumen are found in the urine, and the specific gravity is persistently subnormal.

The above all represent cases which had gone on with varied symptoms, from one to two years without a diagnosis of nephritis having been made.

*Symptomatology.*—Probably the earliest symptom of interstitial nephritis is an increase of the quantity of urine excreted; but this is so gradual, and is so insidious, that the patient's attention is hardly attracted to it until long after other symptoms have developed; in fact, very often it will not have made an impression upon him until the physician makes special inquiry concerning it. This will have been manifest by a desire to empty the bladder earlier than the accustomed time for rising, and as time goes on the patient is obliged to get up earlier and sometimes several times during the night. Gradual loss of flesh, decreasing strength, anorexia, depressed spirits, paleness of the skin and particularly of the mucous membranes are common to all cases. Localized œdema is probably present at some time in every case, but it is not an early symptom and is most frequently found affecting the lower eyelids or the connective tissue above the ankles. It is so slight at times as to be scarcely perceptible, and careful examination for pitting on deep pressure only reveals it. Occasionally œdema occurs in unusual locations, as is shown in one of the above cases, where it appeared in the soft tissues over and about the larynx. There is hardly any region where it does not occur, but it is to be remembered that it differs from œdema occurring in other varieties of nephritis in being transient and regional. Like that in all varieties, it is my experience that it is worse in the morning than at night, a fact which is not commonly mentioned.

The heart is usually enlarged, and the degree of the hypertrophy corresponds to the duration of the disease, and probably to the diffuseness of the inflammation. In this connection the pulse is hard, incompressible, and the radial artery can be felt nearly to the elbow, a condition which represents a change in the arterial wall, which is supposed by its increased resistance to be accountable for the cardiac hypertrophy. Other symptoms have reference to the digestive, respiratory and nervous systems, and are by no means uniform. As a rule, the indications point so stronly to one of these, that in the absence of other well-marked evidences of kidney disease, an error in diagnosis is commonly made, and the patient supposed to have some affection of the stomach, the lungs or the brain.

Probably in most cases the digestive organs appear at fault. Distress after eating, nausea, vomiting, torpor of the liver, at times constipation, and again diarrhœa, are the most frequent. In another class, headache, extreme nervousness, sleeplessness, mental depression, and later convulsions, followed usually by coma, are found; and in still another, attacks of pulmonary œdema and congestion. Bronchitis, asthma or affections of the glottis make up a marked part of the clinical history. There is a peculiarity about any or all of these classes of symptoms, however, which I have learned from observation, and that is their explosive an l intermittent character. This was markedly illustrated in two of the cases above reported; so much so in one that I was inclined to regard the case as one of malaria, until my error was suggested by a failure in the treatment advised. These outbursts are undoubtedly due to an overdose of urea, and hence occur only after the disease has made considerable progress. Eye symptoms, not uncommon in other varieties of nephritis, are frequent in this, but toward the last, when uræmic poisoning is more marked, there is sluggishness of the pupillary reflex, which gives to the eye a vacant expression, and occasionally temporary amaurosis occurs.

The urine always presents indications which are characteristic of this affection, and if intelligent urinalyses were more frequent in the daily investigation of cases, fewer patients would go on to advanced cirrhosis of the kidney unrecognized. The color is pale, amount increased, specific gravity subnormal, and these conditions are constant. Albuminuria may or may not be constant. It is generally slight in amount, and frequently repeated examinations at intervals are essential before it is found. The presence of casts is also variable, and when detected, they are of the hyaline character.

The etiology of interstitial nephritis is somewhat obscure. Opinions differ as to the relation of the ordinary causes given in the books—syphilis, spirit drinking, lead poisoning and hereditary influences—to this disease. Frequently other conditions existing in an individual may be assumed to act as an exciting cause. In two of the cases before referred to, I believed a chronic inflammation of the urethra in one and of the bladder in another to be the cause. In the other two, there was no ascertainable cause, unless it existed in a preceding diphtheria in one and scarlet fever in the other. Could not these diseases in childhood be the cause of an interstitial inflammation of the kidney, which progressed so slowly as not to be recognized until adult life, or could not a parenchymatous inflammation, the result of such a disease, be recovered from and yet start the interstitial inflammatory process? In view of the well-known fact that diphtheria and scarlet fever have a strong affinity for the kidneys, and that interstitial nephritis is not so infrequent before thirty years of age, I

think it reasonable to answer these questions in the affirmative.

The diagnosis is not difficult. The reason for its delay is found in the apparent indifference on the part of the physician to investigate the condition of the urine, and the absence of well defined symptoms pointing to the kidneys as the seat of disease. The patient is always ready to state that his urine is all right, that it is as clear and free as possible, and his medical adviser is too apt to accept this explanation as denoting absence of kidney troubles. In practice, I have come to make urinalyses as frequently as I look over the lungs and heart in examining patients, and in all cases presenting clinical histories not corresponding to recognizable disease or conditions, or in apparently simple troubles not yielding to treatment, my first thought is of the urine. An error which I believe to be widely prevalent, too, is the dependence which is placed upon the presence or absence of albumen in determining an opinion of the condition of the kidneys. A urinalysis for practical purposes is not complete without noting quantity of urine in twenty-four hours, color, re-action, specific gravity, presence or absence of albumen, and without such, one is not justified in reaching a conclusion as to renal disease. The urine must be depended upon in the diagnosis. If then, in a given case presenting any phase of the clinical history already cited, the urine be found constantly increased in amount, of pale color, low specific gravity, to contain albumen in small quantities, occasional or constant, we are justified in making a diagnosis of interstitial nephritis. I would not hesitate to pronounce upon a case which presented only the above urinary conditions, with hypertrophy of the heart, without another symptom; in fact, a low specific gravity and a trace of albumen alone are strongly suggestive, and if constant, a diagnosis would be corroborated by the subsequent history.

This disease should be discriminated from other varieties of nephritis, as the differentation has a marked bearing on the prognosis. Chronic parenchymatous nephritis is attended with constant œdema, oftimes anasarca, the heart is not so constantly enlarged, the urine is not so increased in quantity, has more color, always contains albumen in considerable amount, and the specific gravity is subnormal and may be increased in the early period. The disease comes on more rapidly and runs its course in a shorter time. If prolonged for a number of years, interstitial inflammation is a consequence, and we have a mixed disease which presents more the history of the latter, but the entire clinical history will enable us to determine its commencement in the former. Acute parenchymatous nephritis would hardly be confounded. The urine here is of high specific gravity, contains even a larger amount of albumen and is less than normal in quantity.

*Pathology.*—Many years ago, Gull and Sutton called attention to a general arterial sclerosis which was found in all cases of interstitial nephritis, and after studying carefully many hundreds of autopsies in which these conditions were associated, in some of which there had been no evidence during life of any kidney affection, the patient dying of an intercurrent disease, they maintained that the nephritis was but a local manifestation of a general pathological process having its origin in an inflammation of the intima of the blood vessels; in other words, that the cirrhotic kidney was a constitutional rather than a local disease. They explained, on this theory, the dependence of the cardiac hypertrophy upon the interference with the general circulation occasioned by rigid arteries. Pathologists are still divided as to the correctness of this theory, the opposition maintaining that the arterial sclerosis is the result of chronic uræmia and occurs in other varieties of chronic nephritis, and also, that if the constitutional theory was correct, the inter-cellular tissue of other organs than the kidneys would be equally involved. Certain it is any way that the disease is manifest in an inflammation of the connective tissue structure of the kidney, which leads to contraction and consequent atrophy of the excreting portions. This process may or may not be equally diffused through the organs, hence the variation in the duration of the disease.

*Prognosis.*—As to absolute recovery, the prognosis is most unfavorable, yet the disease may last for many years, and the patient be in apparent good health. Conditions of climate, occupation, habits, so affect the disease for better or for worse that the subjects, so to speak, hold their lives in their own hands. Acute inflammatory diseases, as intercurrent affections, render the prognosis as to time unfavorable, and particularly is this so of acute nephritis which is most liable to precipitate a fatal acute uræmia.

*Treatment.*—Nothing is to be expected in the way of cure, hence all efforts should be directed to retarding the progress of the affection. A kidney, which is the seat of interstitial inflammation, is a permanently damaged kidney, and it should be relieved of its work to the greatest possible degree. The chief indications to this end have reference to limiting the production of urea and its compounds in the economy, and attention to the healthy action of those organs which act supplementary to the kidneys, namely, the skin and bowels. The first indication is accomplished by a restriction of the ingestion of urea-producing foods to the lowest point consistent with good nutrition, and the avoidance of all articles which are not easy of digestion and transformation into tissue-building elements. With these objects in view, it is that milk alone has for many years been selected as the ideal food in kidney diseases. That it has any specific or curative effect, I do not believe. When it can be well borne and taken in sufficient quantities, it certainly answers an excellent purpose, but milk is a much abused food; few patients will admit that they can take it in such quantity as is considered necessary to sustain life. One of mine was quite rebellious until he learned not to drink it, but to sip it, and consumed three-quarters of an hour taking a quart. This he kept up three times daily for nearly a year before he increased his diet, and then he was not anxious to do so.

The second indication is to keep the skin active—constantly active—not by depleting measures, but by the natural method, a warm atmosphere, and this should be as uniform as can be found. I believe the climatic treatment of all forms of chronic nephritis is of greater importance than that of phthisis. Two objects are attained by a warm climate which presents the slightest variations the year round, the production of a constant perspiration and the freedom from the possible danger of sudden chilling of the surface, and I believe it is a mistake for patients who are able to permanently take up an abode in such a place not to do so. Temporary residence only involves climatic changes which are too often productive of harm. The chief indications having been fulfilled, other treatment has reference to management of symptoms which will vary in different cases.

